# Recent Advances in Natural Fibre-Based Materials for Food Packaging Applications

**DOI:** 10.3390/polym15061393

**Published:** 2023-03-10

**Authors:** Harikrishnan Pulikkalparambil, Sandhya Alice Varghese, Vanee Chonhenchob, Tarinee Nampitch, Lerpong Jarupan, Nathdanai Harnkarnsujarit

**Affiliations:** 1 Department of Packaging and Materials Technology, Faculty of Agro-Industry, Kasetsart University, 50 Ngam Wong Wan Rd., Latyao, Chatuchak, Bangkok 10900, Thailand; 2 Center for Advanced Studies for Agriculture and Food, Kasetsart University, 50 Ngam Wong Wan Rd., Latyao, Chatuchak, Bangkok 10900, Thailand

**Keywords:** natural fibres, food packaging, fibre modifications

## Abstract

Packaging is one of the major domains in the food processing industry that reduces waste and enhances product shelf life. Recently, research and development have focused on bioplastics and bioresources to combat environmental issues caused by the alarming growth of single-use plastic waste food packaging. The demand for natural fibres has recently increased because of their low cost, biodegradability and eco-friendliness. This article reviewed recent developments in natural fibre-based food packaging materials. The first part discusses the introduction of natural fibres in food packaging, with a focus on fibre source, composition and selection parameters, while the second part investigates the physical and chemical ways to modify natural fibres. Several plant-derived fibre materials have been utilised in food packaging as reinforcements, fillers and packaging matrices. Recent investigations developed and modified natural fibre (physical and chemical treatments) into packaging using casting, melt mixing, hot pressing, compression moulding, injection moulding, etc. These techniques majorly improved the strength of bio-based packaging for commercialisation. This review also identified the main research bottlenecks and future study areas were suggested.

## 1. Introduction

**Figure 1:**
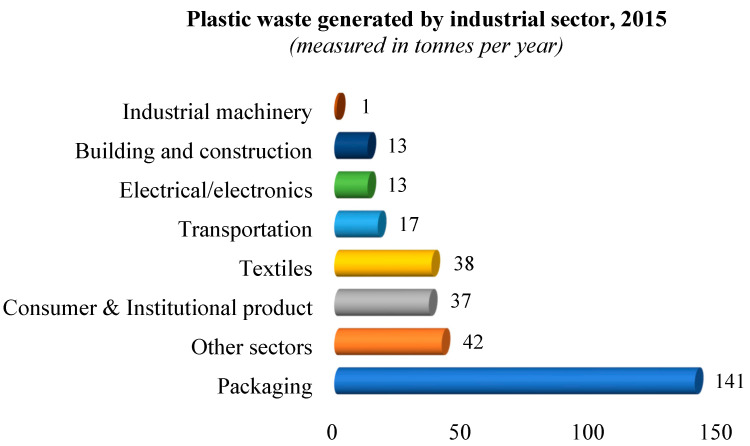
Plastic waste generated by different industrial sectors [2].

Both small- and large-scale food industries are growing continuously, with food packaging being an integral aspect to reduce spoilage and extend product shelf life [1]. The global production of plastics is projected to reach 1100 million tonnes by 2050, with 36% of the output currently used in the packaging industry and 85% of this ends up in landfills. Figure 1 shows a graphical representation of plastic waste generation by several industrial sectors in 2015 [2]. This discarded waste pollutes the environment. Plastic packaging is now used to produce complex geometries with functional snap fits and decorations but single-use plastics cause extreme ecological issues. High production volumes, short usage time and dealing with the disposal of end-life plastics have become pressing issues. Rates of recycling for conventional single-use packaging such as glass, plastic, paper, aluminium, and other alloys are low, with paper and paper-based packaging materials recycled around 20% of the time, while others such as plastic are recycled at substantially lower rates [3].

Packaging material has a vital role in product functionality, efficiency, processing parameters and environmentally friendly customer satisfaction [4,5]. Petroleum-based conventional plastic packaging is mostly non-biodegradable, with rising and unstable prices due to fluctuations in the availability of petroleum sources. Packaging disposal is now a primary concern threatening to pollute water supplies, sewer systems, rivers and lakes [6]. Over time, plastic products fragment into micro- and nano-sized particles that cause serious health issues [7]. Microplastics have been detected in 15 human biological components including breastmilk, bronchoalveolar lavage fluid, blood, lung, liver, kidney, spleen, placenta, meconium, skin, hair, head, face, hand, saliva, colectomy specimens, faeces and sputum [8]. Babies ingest 553 to 4,550,000 microplastic particles/day through feeding bottles [9]. This microplastic exposure directly impacts the digestive, reproductive, central nervous, immune and circulatory systems during early developmental stages.

Pollution caused by plastics requires waste management action by innovation, improved product and package design and increased recycling. This requires organised legislative actions and international cooperation. The statistics indicate that the utilisation of plastic resources in the takeaway food industry has mushroomed [10]. China is the largest consumer of takeaway food, generating 350 kT/day of plastic food packaging, with 40 billion food boxes discarded per day in 2019 [11]. Recently, the COVID-19 crisis caused a 2.2% reduction in the use of plastics in 2020 but the volumes of takeaway packaging and consumption of plastic medical equipment increased as economic activity resumed in 2021. This upward progression of the use and consumption of plastics must be critically addressed [12,13].

This study investigated alternatives to traditional synthetic plastic packaging by adopting a sustainable, renewable and biodegradable approach [14,15]. Natural fibres are commonly used as reinforcement in composite materials [16]. They play pivotal roles in attaining some of the specific needs in composite preparations. Recently, the utilisation of natural fibres has increased because of ecological concerns; they are lightweight, naturally degradable, CO_2_ neutral and readily available as renewable materials [17,18]. Most importantly, due to their vulnerability to living organisms, they are biodegradable and do not impact the ecosystem [19]. Consequently, incorporating natural fibres into the polymer matrices improves the disposal of composite material [20,21,22]. Varghese et al. [23] investigated the use of *Ceiba pentandra* natural fibres in poly(3-hydroxybutyrate-co-3-hydroxyvalerate)-based packaging applications. They found that the incorporation of natural fibres accelerated the degradation of packaging materials, which showed good antibacterial capacity against *Staphylococcus aureus* and effectively preserved the freshness of strawberries for a longer period. Natural fibres are ecologically friendly but negative packaging aspects include their dominant hydrophilicity and low mechanical properties under humid conditions. Therefore, uses of natural fibres in several packaging applications are limited. Recently, natural fibres have been utilised when the recovery of conventional plastics is not economically feasible, controllable or viable and one-time-use packaging materials are preferrable. Natural fibre-reinforced composites can be reused, unlike cardboard boxes. Saraiva et al. [24] developed natural fibre-reinforced composite material from sponge gourd residue and compared its packaging efficacy with that of cardboard boxes. The results showed that the developed natural packaging material was preferable after four cycles of use. 

This review aimed to investigate advancements in the research, development and utilisation of natural fibre-based composites for food packaging applications. Sources, compositions and recent production techniques of several natural fibres in food packaging were discussed. Physical and chemical modifications of natural fibres that improve their suitability for food packaging were also explored.

## 2. Natural Fibres in Packaging

Natural fibres are abundantly available as biodegradable and renewable natural materials [25] and they have recently received huge attention from the global research community [26,27]. Natural fibres can be divided into three categories by origin: animal-based, mineral based and plant-based [28]. Plant-based natural fibres were the main focus of this review because of their abundant availability at low cost. Plant-based natural fibres are lignocellulosic in nature with their basic constituents including lignin, hemicellulose and cellulose. Animal-based fibres mostly consist of proteins, e.g., wool and silk. Mineral-based fibres are formed as a result of geological processes, such as asbestos and basalt. In plant-based natural fibres, both primary fibres obtained directly from plants and secondary fibres obtained as byproducts after utilisation of primary fibres are used.

### 2.1. Source and Compositions

**Figure 2:**
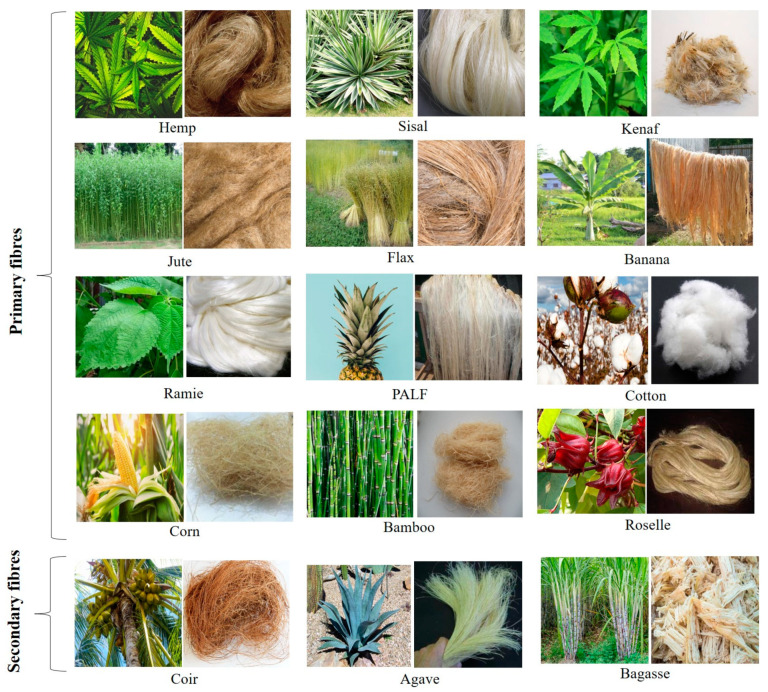
Commonly available natural plant fibres and their sources [32,33,34,35,36,37,38,39,40,41,42,43,44,45,46,47,48,49,50,51,52,53,54,55,56,57,58,59,60] Agave fiber figure Reprinted with permission from Ref. [58]. Copyright 2021 Elsevier Ltd.

Natural primary plant fibres include hemp, kenaf, sisal and cotton, while secondary fibres include bagasse, coir, pineapple, agave and oil palm [29,30,31]. Natural fibres have long been exploited in the preparation and manufacture of ropes and textiles, for example, flax, hemp, cotton lint or sisal. Some fibres have secondary applications in food packaging. Figure 2 shows some of the commonly available natural fibres and their sources, while Table 1 shows the origin and properties of natural fibres. Some of the major varieties of natural fibres are discussed in more detail below.

#### 2.1.1. Hemp (*Cannabis sativa*)

Hemp is one of the most widely utilised natural fibres after sisal as reinforcement for composites [61]. Hemp is grown widely in the EU, China, the Philippines and Central Asia. The plants are cultivated from seed and can grow up to 5 m in height. Crops cultivated for fibre are densely sown and produce plants averaging 2–3 m in height with almost no branching. Hemp fibres have antibacterial properties [62,63,64] emanating from cannabinoids, alkaloids, other bioactive components or lignin [65]. Khan et al. [66] studied the antibacterial properties of hemp hurd powder against *E. coli* using retted, semi-retted and non-retted hemp hurd powder with different particle sizes. The fibres were kept at 160 °C for 2 h to eliminate self-contaminations such as humidity and thermal history. These authors found that hemp was an ecofriendly food packaging material suitable for meat, salads and ready-made food products. Teixeira et al. [67] studied the temperature effects on mechanical strength of hemp fibres. They found that tensile strength increased by 18% once the fibres were exposed to 100 °C for 24 h. However, when exposed to 200 °C for 24 h, tensile properties decreased and the fibres became fragile and brittle.

#### 2.1.2. Sisal (*Agave sisalana*)

Sisal is one of the most commonly used natural fibres grown in tropical and subtropical regions of North and South America, Africa, the West Indies and the Far East. Moreover, the hydroalcoholic extract obtained from sisal leaves possesses significant antimicrobial activity against *Aspergillus niger* and *Candida albicans* [68,69]. Pulikkalparambil et al. [70] examined the reuse of discarded polypropylene (PP)-based disposable face masks with sisal and hemp fibre mats. They used hot compression moulding to sandwich the PP masks and natural fibres. The resulting composites showed excellent mechanical properties with antimicrobial activities against *S. aureus*.

#### 2.1.3. Kenaf (*Hibiscus cannabinus* L.)

Kenaf is a member of the Malvaceae family and herbaceous fibre crops. Kenaf plants are grown throughout the year with a short harvest time in West Africa, India and China [71]. They can grow up to 2.5–4.5 m tall with stems up to 1–2 m in length. Kenaf seeds and leaves are used in food products as they are rich in nutritional and phytochemical compounds [72].

#### 2.1.4. Bamboo (*Bambusa vulgaris*)

Bamboo (*Bambusa vulgaris*) grows in Asia-Pacific, African, European and North and South American regions. Bamboo reaches maturity in 3 years when its tensile strength is effectively comparable to mild steel. Moso bamboo has a growth rate of 2 inches per hour. Some bamboo species reach a height of 60 feet in 3 months. Therefore, cutting down this wood does not affect the ecological and natural balance much [73]. Bamboo is considered the most under-utilised natural fibre and is abundantly available in Southeast Asian countries. The annual global availability of bamboo fibres is 30 million tonnes with a maturity cycle of only 3–4 years. Bamboo fibres have excellent mechanical strength. The specific stiffness and strength is comparable to glass fibres [74]. Afrin et al. [75] reported strong antibacterial properties of bamboo fibres against *E. coli* and *S. aureus* due to retained lignin. Another possible reason was the presence of H_2_O_2_ that damaged the DNA sequencing of *E. coli*. However, they believed that this was not possible as H_2_O_2_ was thermally decomposed during the extraction process.

#### 2.1.5. Jute

Jute is often neglected but considered as one of the most important fibres. Jute is in the Tiliaceae family with the scientific name Corchorus capsularis because it is extracted from Corchorus plants. Jute fibres are mostly found in the Mediterranean but recently the finest growth fibres come from Bangladesh, India, China, Nepal, Thailand, Indonesia and Brazil. Jute fibres are brittle and can grow 2–3.5 m in height. They possess very high lignin content (12–16%) and thus have low elongation at break. Jute fibres possess unique properties which, if utilised effectively, can solve problems in the textile and food packaging fields. The incorporation of jute fibres in PLA matrices improved both oxygen and water vapour barrier properties.

#### 2.1.6. Flax (*Linum usitatissimum*)

Flax is commonly grown in moderate climatic regions such as India, Argentina, Southern Europe, China and Canada [28]. Flax plants can grow to heights of 80 to 150 cm in less than 110 days. Fibres from flax bast grow between 60 and 140 cm long with diameters ranging from 40 to 80 µm. They are members of the bast family. The bast fibres are collected from the fibrous bundles located in the inner bark of a plant stem. The major components of flax fibres are pectin, hemicellulose, cellulose, and lignin. There are also small amounts of wax, oil and water. The incorporation of flax fibres increases strength and stiffness, which can be further improved by modification with a malleated coupling agent [76].

#### 2.1.7. Banana Plants

Banana plants are mostly grown in tropical countries where they are considered as an agricultural crop. Banana bast fibre is a lingo–cellulosic material and extracted as a waste product of banana plant cultivation. Banana fibre has great specific strength which is comparable to conventional materials such as glass fibre [77]. Rana et al. [78] manufactured banana fibre-reinforced polyvinyl alcohol (PVA) resin and evaluated the mechanical strength of these composites. They concluded that PVA composites with reinforced banana fibre could be used as biodegradable food packaging. They had good biodegradation with adequate handling strength.

#### 2.1.8. Ramie (*Boehmeria nivea* (L) Gaud.)

Ramie is a perennial hardy shrub belonging to the Urticaceae family. Ramie is considered one of the oldest vegetable fibres which has been utilised for thousands of years, specifically as mummy cloths in Egypt from 5000–3000 BC. Ramie was initially grown in China, while today ramie fibre is mainly grown in Brazil, India, China, the Philippines, South Korea, Taiwan and Thailand. The fibres are popularly known as Rhea, Kunkura, Pooah, Kunchoor, Puya, steel wire and China grass in different parts of India. There is high demand for ramie fibres due to their performance and aesthetic properties. Ramie fabrics effectively absorb moisture, transmit heat, and are more resistant to mildew than other cellulose-based fibres.

**Table 1:** Origin and properties of natural fibres. Reprinted with permission from Ref. [79]. Copyright 2021 Wiley Ltd.

Natural Fibre	Origin	World Production (×10^3^ Tonnes)	Density (kg/m^3^)	Diameter (μm)	Tensile Strength (MPa)	Tensile Modulus (GPa)	% Elongation
Abaca	Leaf	70	0.83	114–130	418–486	12–13.8	-
Banana	Stem	200	1.35	80–250	529–759	8.20	1–3.5
Bamboo	Stem	10,000	910	-	503	35.91	1.4
Coir	Fruit	100	1.15	100–460	108–252	4–6	15–40
Cotton	Lint	Fruit 18,500	1.6	-	287–597	5.5–12.6	3–10
Flax	Stem	810	1.5	-	345–1500	27.6–80	1.2–3.2
Jute	Stem	2500	1.46	-	393–800	10–30	1.5–1.8
Hemp	Stem	215	1.48	-	550–900	70	1.6
Kenaf	Stem	770	1.4	81	250	4.3	-
Oil palm	Fruit	Abundant	0.7–1.55	150–500	80–248	0.5–3.2	17–25
Ramie	Stem	100	1.0–1.55	20–80	400–1000	24.5–128	1.2–4.0
Rice husk	Fruit/ grain	Abundant	-	-	-	-	-
Roselle	Stem	250	-	-	-	-	-
Sisal	Leaf	380	1.45	50–300	227–400	9–20	2–14

### 2.2. Natural Fibre Selection Parameters as Packaging Material

The function of food packaging is to protect the food contained inside the packaging from physical, chemical and biological hazards (oxygen, moisture, light, microbial contamination and insects) [80]. Packaging materials depend greatly upon the type of food. contained inside the packaging such as meat, fruits, vegetables and ready-to-eat food. The packaging must maintain the safety and quality of the contained food. Other functions including proper containment, convenience, and information regarding the food are required, while most importantly the packaging must look aesthetically pleasing. Table 2 lists some of the categories investigated when studying the properties of packaging materials [81].

One primary concern is the structural aspects of the packaging material including tensile and tear, strength, bending, compression, puncture and folding parameters that need to withstand different loading conditions during stacking, transfer and transportation. The strength of natural fibre-reinforced composites relates to two factors: (a) the stiffness and strength of the natural fibres and (b) compatibility between the fibres and the matrix. Strength and stiffness depend on the arrangement of cellulosic fibrils in the microfibrils present in the fibres, while the mechanical properties of natural fibres are contingent on the part of the tree or plant from which the fibre has been collected. Crystalline and amorphous fibre characteristics differ between parts of the tree and between trees. Previous studies suggested that fibres with fewer amorphous contents such as hemicellulose, lignin and pectin possess better mechanical properties [82,83].

The concentration of natural fibres incorporated into composites also impacts the mechanical properties. An increase in fibre content increases mechanical properties up to an optimal concentration beyond which the mechanical strength starts to show a decrease trend [84]. Tao et al. [85] studied the effect of jute and ramie fibre loading in PLA composites and found that 30% natural fibre content in PLA provided the optimal mechanical properties. Another factor affecting the properties of natural fibre-reinforced composites is compatibility, where interaction between the fibres and the matrix plays an important role in uniformly distributing the applied load into the matrix. Kamarudin et al. [86] reported that PLA/kenaf composites showed excellent mechanical strength at up to 40% fibre loading due to good fibre–matrix interfacial interaction. Beyond this critical fibre loading value, poor filler matrix compatibility resulted in earlier fracture of the composite. Several surface modification techniques (both physical and chemical) have been studied to improve the compatibility of natural fibres and matrix materials.

Composites prepared from natural fibres show promise applied as food packaging materials. However, the role of food packaging materials is not limited to protecting products from physical and mechanical damage during distribution [87]. Food packaging must also control the transfer of water vapour, oxygen and/or carbon dioxide, which impact rates of oxidation, microbial development and physiological reactions of food degradation. Plastics are commonly permeable to small volatiles such as gases (O_2_, CO_2_), water vapour, organic vapours and liquids [88,89] and water absorption barrier properties are essential basic requirements when packing food. The moisture barrier property is important in food packaging as this preserves the texture in both dry and moist food and controls microbial growth of aerobic spoilage. Important parameters responsible for the control water vapour permeability (WVP) are fibre content and size, fibre/matrix adhesion and crystallinity and plasticisation of the matrix [90,91,92]. The dispersion of fibres in the matrix can evoke impermeability due to the tortuosity effect. However, the WVP of natural fibre-reinforced composites significantly increases due to the hygroscopic nature of the fibres and poor dispersion in the matrix. By contrast, hydrophilic polysaccharide matrices have low WVP properties. Sirvio et al. [93] observed that incorporation of up to 50 wt% of cellulose microfibrils in alginate films decreased the WVP due to an increase in tortuosity. However, the aggregation and percolation of small natural fibres in polymer matrices can result from poor fibre/matrix adhesion, leading to voids in the polymers which encourage the transport of water molecules [94]. Other ways to improve the WVP of natural fibres include coating with PLA [95]. Das et al. [96] observed enhanced vapour permeability and absorption capacity of 15.9% and 48.1%, respectively, in shredded betel nut composite sheets compared with cardboard sheets. Food must also be protected from oxygen and carbon dioxide, which cause many degradation reactions. High levels of CO_2_ in chilies limit the Krebs cycle, whereas low levels of O_2_ decrease the activation of cytochrome oxidase, polyphenol oxidase, glycolic acid oxidase and ascorbic acid oxidase [97]. 

Migration into food is another parameter to be considered when choosing materials for food packaging applications [98]. Toxicological substances such as pesticide residues such as herbicides, fungicides and insecticides and other pollutants in the environments such as polycyclic aromatic hydrocarbons might be present in natural fibres. Special additives such as plasticisers, including phthalates, are also added in formulations of packaging materials. These unnatural substances might migrate into food during storage causing spoilage. Temperature, activation energy and microstructures of the packaging films can also restrict diffusion of unwanted substances into food [99,100]. Thus, it is necessary to decontaminate natural fibres during extraction before composite preparation.

Finally, the biodegradability/compostability of the packaging materials is also of interest to consumers. Using full bioplastics as matrices and natural fibres produces eco-friendly packaging with low carbon footprints. Biodegradation rates of natural fibre composites depend on the nature of the fillers, reinforcements and the matrix as well as the composite ratios.

**Table 2:** Properties of packaging materials.Reprinted with permission from Ref. [81] Copyright 2008 Elsevier Ltd.

Property	Examples
Structural properties	Tensile strength, tear properties, compression properties, bending stiffness, edge crush resistance, burst strength, puncture resistance, folding endurance, wet strength and delamination
Barrier and absorption properties	Oxygen permeability (OP), water vapour permeability (WVP), Volatile permeability and water absorption capacity
Manufacturability and manufacturing quality	Uniformity of thickness, density and moisture content
Migration into food	Toxicology parameters and migration studies
Non-structural functionality	Abrasion resistance and static and kinetic friction
Degradability/compostability	Compostability in biodegradation tests and disintegration tests

## 3. Physical and Chemical Modifications of Fibres for Food Packaging

Numerous studies have investigated natural fibre-reinforced polymers because of their improved strength and stiffness with the low cost, biodegradability, renewability and abundancy of natural fibres [101,102]. However, mixing natural fibre with a polymer matrix commonly causes poor mechanical composite properties because of (a) poor compatibility between the polar hydrophilic natural fibre and the non-polar hydrophobic polymer matrix and (b) non-homogeneous dispersion of fibre and wood powder in the PP matrix [103,104]. The modification of natural fibres can reduce their hydrophilicity and, thereby, increase their compatibility. The removal of unwanted wax and increasing surface roughness increases the contact surface area of the fibres, which in turn increases the transfer of stress uniformly into the matrix. Several chemical and physical treatments to improve the compatibility of natural fibres are as follows.

### 3.1. Chemical Modification Techniques

Composites reinforced with chemically treated natural fibres generally show enhanced mechanical properties because of improved interfacial adhesion between the fibre and the matrix [82]. Numerous surface treatment methods are available for the modification of natural fibres. Alkaline (NaOH) treatment/mercerisation is the simplest, most effective and commonly used chemical treatment. Traditionally, mercerisation is a technique to modify surface of cotton. Strong caustic soda solutions are used to treat materials for 1–3 min under tension and low temperatures, followed by washing. Holding cotton fabric under tension in the caustic solution helps to maintain its original dimensions. As a result, the fibres have more a rounded structure in the cross section, reflecting light to improve lustre. Mercerised cotton also involves a change in crystalline structure and degree of crystallinity, thereby reducing stresses and increasing the strength of the weak points in the fibre [105].

Immersion in NaOH aqueous solution removes hemicellulose, lignin, pectin and other impurities from the fibre surface. This results in a rougher surface, which in turn improves the mechanical interlocking of the fibres with the matrix. Borah et al. [106] found that alkali treatment of betel nut fibres before composite formation improved tension strength by 18%, elongation at break by 6%, bending strength by 11% and impact strength by 18%. The reaction between the fibre and alkali solution can be represented by the equation below [107].

$$\mathrm{Fibre}-\mathrm{OH}\;+\;\mathrm{NaOH}\;\to \mathrm{Fibre}-O-{\mathrm{Na}}^{+}+H_{2}O\;$$

The utilisation of oxidising agents such as hydrogen peroxide (H_2_O_2_) in natural fibres can also eliminate the cementing substances from surface of the fibres, which hinder adhesion with the polymer matrix. For natural fibres, alkali treatment results in the formation of an alkali-resistant linkage between lignin and hemicellulose that may impede the removal of lignin. Using H_2_O_2_ breaks these bonds and delignifies lignocellulosic fibre, which enhances the interfacial adhesion with the polymer matrices [108,109]. The acetylation of fibre also improves hydrophobicity, which enhances interfacial adhesion by reducing the moisture absorption of the cellulose components. This includes treatments of acetic or propionic acid at elevated temperatures with or without the combination of an acid catalyst [110]. Acetylation of the cellulose components substitutes hydroxyl groups of the cell wall, which increases the hydrophobicity of the natural fibres. This method improves compatibility with the polymer matrix by decreasing water absorption.

Finally, coupling agents also reduce inherent incompatibility between the polymer matrix and natural fibres, which enhances the interfacial adhesion. Polymers consist of bifunctional groups which effectively react with both the fibre and the matrix. Organofunctional silane coupling agents form covalent bonds with the hydroxyl groups of cellulose. Alkoxy groups are hydrolysable. Moisture facilitates hydrolysis and forms silanols which further react with the hydroxyl groups of the fibre. Consequently, stable covalent bonds are formed with the cell wall that are chemisorbed onto the fibre surface [110,111,112]. This chemisorption commonly improves the degree of cross linking at interface, which improves affinity of organophilic polymers [110]. Adding hydrocarbon chains by the modification of natural fibre with silanes modifies their wettability and reduces water uptake as covalent bonding forms cross-linking between the fibre and the matrix.

However, the use of alkaline or any other chemical treatments adversely impacts product sustainability, with green techniques preferred for natural fibre modification. Smith et al. [113] prepared a sustainable green composite based on agave fibre (*Agave tequilana*) modified with poly(3-hydroxybutyrate) (PHB) in the presence of a small quantity (0.1 phr) of organic peroxide through one-step reactive extrusion processing. Results showed that 25 wt% agave fibre with 0.1 phr peroxide improved flexural strength by 46%, impact strength by 45% and heat deflection temperature (HDT) by 39% compared with neat PHB. These findings suggested that the presence of peroxide provides a cost-effective and sustainable alternative to petroleum-based conventional plastics for food packaging.

Mohanty et al. [114] chemically modified date palm leaf (DPL) using acrylic acid and tested the dispersion and compatibility with polyvinylpyrrolidone composites for packaging applications. Prepared biocomposites reinforced with 26 wt% DPL fibre loading showed promise for use as water- and chemical-resistant hydrophobic packaging materials. Nazrin et al. [115] studied the incorporation of nanocellulose to enhance the properties of thermoplastic starch (TPS), polylactic acid (PLA) and polybutylene succinate (PBS) for food packaging. They reported that the addition of nanocellulose in TPS improved the low water barrier and tensile properties, while the addition of nanocellulose into PBS and PLA enhanced the oxygen barrier properties and mechanical strength.

Natural jute fibre incorporated with a red grape pomace extract (RGPE) has been developed for active packaging. The RGPE was derived from pressurised liquid extraction (PLE) and enhanced solvent extraction (ESE) techniques [116]. The packaging showed excellent antibacterial activities against *E. coli*, *S. aureus* and *Pseudomonas aeruginosa*. The RGPE extract from PLE using C_2_H_5_OH:H_2_O (as a solvent) had 11 major phenolic compounds. Jara-Palacios et al. [117] found quercetin-3-O-glucoside as the most abundant compound in RGPE extracted by C_2_H_5_OH:H_2_O. Wang et al. [118] modified hemp fibre with a lysine-grafted N-halamine organic as an antibacterial agent in hemp fibre using a mild Schiff base reaction. The materials totally eliminated *Staphylococcus aureus* and *Escherichia coli* in 5 min, while the inhibition zone increased to 18.4 mm.

### 3.2. Physical Modification Techniques

Previous studies mainly focused on the effects of coupling agents and compatibilisers to tailor the mechanical properties of natural fibre-reinforced composites. Most chemical treatments were successful and resulted in increased thermal and mechanical properties. However, some major problems associated with chemical treatments are the high cost and pollution from the disposal of the chemicals after treatment [119]. Plasma treatment introduces functional groups onto natural fibres that form strong covalent bonds with the matrix, leading to a strong fibre/matrix interface. Plasma treatment is simple, short-duration, consumes little energy, and low cost. The technique requires no water or any potentially hazardous chemicals. Surface etching improves the surface roughness of natural fibres and results in better interfacial interaction with the matrices through mechanical linking [120,121,122,123,124]. 

#### 3.2.1. Cold Plasma Treatments

**Figure 3:**
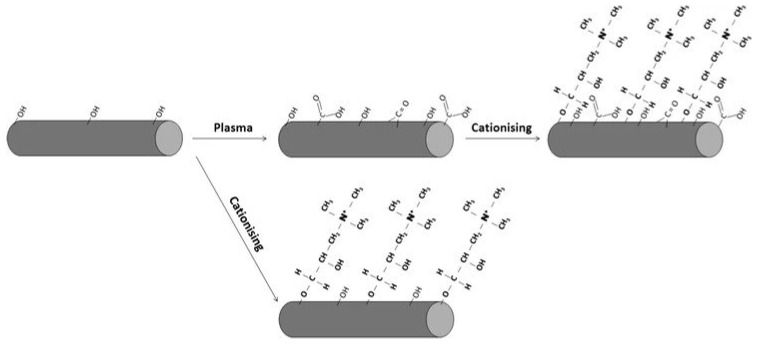
The effects of plasma and cationising processes. Reprinted with permission from Ref. [125]. Copyright 2011 Springer Nature Ltd.

**Figure 4:**
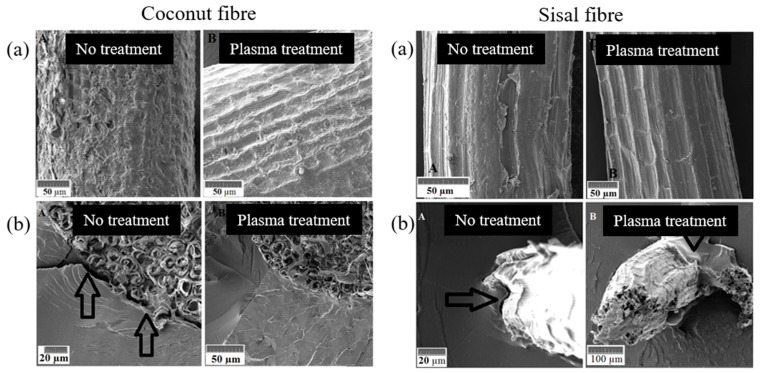
The effect of plasma treatment on (**a**) the surface roughness of fibres and (**b**) the interfacial interaction between the fibre and matrix [126].

Cold plasma techniques are dry, clean processes with less environmental concerns. Such a modification occurs only on the surface with no interference on the bulk properties. Figure 3 shows a schematic representation of the effects of plasma and cationising processes of cellulose-constituting cotton fibres [125]. Sinha et al. [122] studied the influence of physical treatment on the morphology, wettability and impact of the fine structure of fibres on interfacial adhesion of natural fibre-reinforced composites. They found that plasma treatment reduced fibre hydrophilicity due to the decrease in phenolic and secondary alcoholic groups and oxidation of the basic structural lignin and hemicelluloses components. Plasma treatment improved fibre/matrix adhesion, as revealed by scanning electron microscopy (SEM) morphology. Figure 4 demonstrates the etched surfaces and increased numbers of new oxygen functional groups present on the surface of sisal and coconut fibres revealed using SEM analysis [126].

Combining chemical treatments with physical plasma treatments was studied on flax fibres by Gieparda et al. [127] to understand the synergistic effects. The authors used silanisation and plasma treatment both individually and in combination. The results revealed an increased thermal stability with a significant impact on fibre diameter and specific surface area. Erwin et al. [128] studied liquid plasma treatment on coir fibre with microwave plasma in the liquid. The mediums were water and sodium bicarbonate (NaHCO_3_) solution. The interfacial shear strength of the coir fibre–epoxy matrix increased after liquid plasma treatment with both water and sodium bicarbonate because of chemical adhesion which facilitated mechanical interlocking.

#### 3.2.2. Steam Explosion

Steam explosion is another physical modification technique for natural fibres. This involves heating the fibres at a high temperature and pressure, causing mechanical disruption of the cellular material that undergoes fibrillation. The selection of steaming temperature and exposure time is very important to achieve optimal fibre properties. Han et al. [129] studied the effects of steam treatment on wheat straw under different pressures and times. They reported that the treatment enhanced the dimensional stability with the removal of lignin, ash and extracts. 

## 4. Production Technology

**Figure 5:**
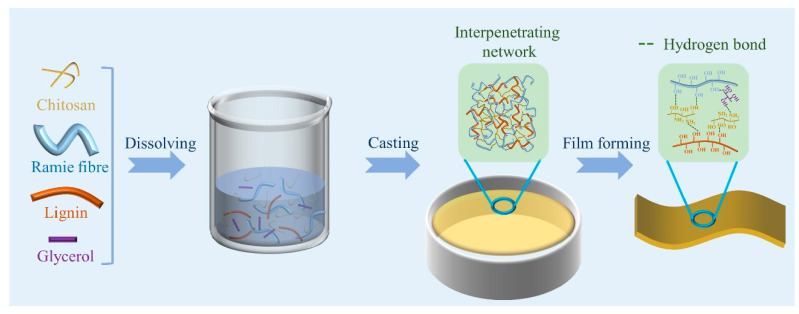
Preparation of chitosan/ramie fibre/lignin composite films. Reprinted with permission from [139]. Copyright 2022 Elsevier Ltd.

Natural fibre-reinforced hybrid composites are now extensively applied to deal with technological problems [130,131,132]. Table 3 lists composites made of natural fibres that have widespread applications in areas where the cost of reinforcements limits the utilisation of conventional, lightweight, reinforced plastic materials [133,134,135]. Cabedo et al. [136] compared almond shell, rice husk and seagrass as fillers in PHB/fibre composites prepared by the melt-blending process. They studied the influence of fibre type and fibre content on morphology, thermal, mechanical and barrier properties, compostability and processability. They concluded that all three fibres were suitable for the development of fully compostable biocomposites for packaging applications. Rawi et al. [75] studied the effects of compression moulding parameters on the mechanical properties of bamboo fabric, poly (lactic acid) (PLA) composites for packaging applications. They reported that the composites with the highest compression pressure of 1.01 MPa at 3 min exhibited a superior tensile strength of 80.71 MPa and flexural properties of 124 MPa. They [137] also compared polypropylene (PP) and bamboo fabric PLA composites to investigate the use of environmentally friendly composites for packaging applications. The findings indicated that bamboo fabric/PLA composites enhanced PLA impact strength by 117%, with comparatively lower impact strength observed for PP/bamboo fibre composites. Thermal stability in terms of the heat deflection temperature (HDT) of PP and PLA matrices was increased by the addition of bamboo fabric. The high heat resistance property of composites is suitable for packaging applications. Nabels-Sneiders et al. [138] studied lamination technology of cast hemp paper with bio-based plastics using a compression moulding process to replace conventional plastics and solve the existing waste disposal problems. They compared polyhydroxyalkanoate (PHA), polylactic acid (PLA), polybutylene succinate (PBS) and polybutylene succinate adipate (PBSA) laminates prepared at three different compression pressures. The desired pressure on porous cast paper, impregnation and excellent layer adhesion was proposed in their study. Ji et al. [139] prepared chitosan-based composite films reinforced by ramie fibre and lignin, as shown in Figure 5, for food packaging applications. The addition of 20% ramie fibre and 20% lignin improved the mechanical properties and water resistance by up to 29.6% and 41%, respectively. Food packaging studies showed extended shelf life in meat products such as chicken breasts compared with fruits such as cherry tomatoes.

Tawakkal et al. [140] used a thymol extract with kenaf fibres to study the migration of thymol extract from PLA/kenaf composites. Melt-blending was used to prepare the films in an internal mixer (155 °C for 8 min and 50 rpm) followed by heat pressing. The materials were melted by preheating (150 °C for 3 min) without applying pressure and then pressed at the same temperature for 2 min with a force of 20 kN before quench cooling to 30 °C under pressure. Tawakkal et al. [141] also studied the antimicrobial activity of PLA/kenaf/thymol against *Escherichia coli* bacteria and naturally occurring fungi. Films with higher thymol concentrations and higher kenaf loading exhibited excellent antibacterial properties against fungal growth due to the release of thymol into the headspace surrounding the samples; however, the shelf life after storage for 3 months at ambient temperature showed only a slight decrease in antimicrobial properties.

**Table 3:** Summary of natural fibre-reinforced composites for food packaging applications.

Source of Fibre	Part of Plant	Fibre Preparation/Treatment	Type of matrix/Other Polymer Blend (If Any)	Role of Fibre in Packaging	Packaging Form	Method of Packaging Production	Major Findings	Ref.
Hemp	Straw	Sodium hydroxide (NaOH) treatment	PHA, PLA, PBS, PBSA	Filler	Paper	Direct melt coating	Biodegradation in a controlled compost at 58 °C resulted in full degradation within 40 to 80 days, with PLA and PHA laminates showing 40 and 50 days, respectively.	[138]
Oil palm	Empty fruit bunch	-	Oil palm empty fruit bunch + Formaldehyde	Matrix	Tray	Solvent casting	Oil palm empty fruit bunch fibre-based trays were below the allowable limit specified by Commission Regulation (EU) No 10/2011.	[142]
Betel nut	Seed	-	Polyester resin	Reinforcement	Laminate	Casting	The resin had favourable characteristics in terms of elasto–plastic and stress–strain behaviour, suitable for storage and transportation.	[96]
Kenaf	Bast	Alkaline treatment	PLA	Reinforcement	Film	Melt blending and heat pressing	Adding kenaf filler to the PLA enhanced the release of thymol from the PLA matrix, reduced production costs and increased mechanical strength. The composite films reduced *Escherichia coli* inoculated on the surface of processed sliced chicken samples after 30 days at 10 °C both in direct contact and in the vapour phase.	[140,141]
Plantain pseudostem	Stem	Acetylation treatment	Polyester	Filler	Laminate	Casting	Flexural strength improved by 28% after acetylation treatment.	[143]
Sugar palm	Trunk	-	Sugar palm + glycerol and sorbitol	Matrix	Film	Solution-casting technique	The introduction of plasticisers reduced brittleness and enhanced flexibility and peelability of films.	[144]
Wheat straw	Straw	-	PHBV	Filler	Film	Heated hydraulic press	A 3.5-fold increase in water vapour permeability was recorded.	[145]
Bamboo	Stem	-	PLA	Reinforcement	Laminate	Film-stacking and compression moulding	The impact strength was enhanced by 117%.	[137]
Date palm	Leaf fibre	Acrylic acid	Polyvinylpyrrolidone	Reinforcement	Laminate	Melt mixing fabrication technique	Biocomposites reinforced with 26 wt% DPL fibre loading can be used as water- and chemical-resistant packaging materials due to their hydrophobic nature.	[114]
*Sterculia urens*	Stem	Alkali treatment and silane-coupling agent	poly (lactic acid) (PLA)	Reinforcement	Laminate	Hot pressing	Alkali treatment in the presence of a silane-coupling agent caused matrix skin formation and the formation of flower-like structures on the surface of the fabric, suggesting good bonding between the reinforcement and the matrix.	[146]
Bamboo	Stem	-	PLA	Reinforcement	Laminate	Film-stacking and compression moulding	The highest compression pressure of 1.01 MPa at 3 min exhibited a superior tensile strength of 80.71 MPa and flexural properties of 124 MPa.	[74]
Coir	Shell fibre	-	Starch/EVOH/Glycerol	Filler	Laminate	Injection moulding	Size and shape irregularities of the fibres played a dominant role in the ultimate properties.	[147]

## 5. Conclusions and Future Perspectives

In the last decade, numerous studies have been conducted on the utilisation of natural fibres to replace conventional polymer applications. Several fibres have been extracted from plant resources such as hemp, sisal, kenaf, bamboo, jute, flax, banana and ramie. Chemical extractions and treatments including alkaline solutions, oxidising agents and coupling agents have been demonstrated to purify and improve the strength of fibre. Alternatively, physical treatments such as cold plasma treatments and steam explosion enhance the properties and purifications of extracted fibre, removing lignin, ash and other substances while increasing dimensional stability. These fibres have been recently utilised in food packaging as matrices, fillers and reinforcements by solution casting, melt mixing, hot pressing, compression moulding, injection moulding, etc. The incorporation of these natural fibres effectively improved the mechanical strength of the packaging. Further development of natural fibre-based food packaging materials can be proposed as follows:

- The valorisation of natural fibres in the food packaging sector exhibited promising results. However, a long-lasting supply of raw materials is essential to ensure sustainability.
- Environmentally friendly extraction/purification is ideal for the production of uniform-quality fibres. The modification of natural fibres needs to address environmental issues implied by chemical methods.
- Natural fibres ensure the safety and protection of food by enhancing the mechanical properties of food packaging to resist physical damage. However, several other factors must be considered. The packaging must be designed to overcome degradation reactions and also be able to regulate gas and water barrier properties. The selection of natural fibres combined with the use of appropriate modification methods can prevent the formation of defects that would degrade the mechanical properties, while also enhancing packaging permeability.
- The decontamination of natural fibres should comply with the regulations on food contact materials to guarantee the health of the consumer. This aspect is challenging when using natural fibres due to the presence of toxicological substances such as pesticides that could migrate to food from the packaging materials.
- Consumer willingness to purchase economically competitive fully biocomposite alternatives is still uncertain. The cost of biocomposites in food packaging materials needs to be regulated to improve the demand in local markets. The future use of natural fibres is highly recommended for packaging materials due to their cost-effectiveness and availability throughout the year.

## Data Availability

Not applicable.
